# Comparative assessment of therapeutic safety of norcantharidin, *N*-farnesyloxy-norcantharimide, and *N*-farnesyl-norcantharimide against Jurkat T cells relative to human normal lymphoblast: A quantitative pilot study

**DOI:** 10.1097/MD.0000000000004467

**Published:** 2016-08-07

**Authors:** Ming-Che Chang, Jin-Yi Wu, Hui-Fen Liao, Yu-Jen Chen, Cheng-Deng Kuo

**Affiliations:** a Laboratory of Biophysics, Department of Medical Research, Taipei Veterans General Hospital, Taipei; b Department of Microbiology, Immunology and Biopharmaceutics, College of Life Sciences; c Department of Molecular Biology and Biochemistry, National Chiayi University, Chiayi; d Department of Radiation Oncology, Mackay Memorial Hospital, Taipei; e Chest Medicine and Physiological Signals Research Center, Changhua Christian Hospital, Changhua, Taiwan.

**Keywords:** 4-parameter logistic model, human normal lymphoblast, Jurkat T cell, NC15, NCTD, NOC15, safety index

## Abstract

The therapeutic safety of an anticancer drug is one of the most important concerns of the physician treating the cancer patient. Half maximal inhibitory concentration (IC_50_) and hillslope are usually used to represent the strength and sensitivity of an anticancer drug on cancer cells. The therapeutic safety of the anticancer drug can be assessed by comparing the IC_50_ and hillslope of anticancer drugs on cancer cells relative to normal cells. Since there are situations where “more anticancer activity” implies “more toxicity,” the safety of an anticancer drug in these situations is hard to evaluate by using IC_50_ and hillslope alone. In a previous study, the “net effect” index was devised to represent the net therapeutic effects of one anticancer drug relative to the other. However, the therapeutic safety of one specific anticancer drug alone was not defined in the “net effect” index. This study introduced the “safety index (SI)” to quantify the degree of safety of an anticancer drug by using 4-parameter logistic model on cancer cells relative to normal cells. The therapeutic safety of norcantharidin (NCTD), *N*-farnesyloxy-norcantharimide (NOC15), and *N*-farnesyl-norcantharimide (NC15) in the treatment of Jurkat T cells relative to human normal lymphoblast was compared using the newly defined SI. We found that the SI of NOC15 and NC15 was significantly higher than that of NCTD, suggesting that both NOC15 and NC15 can damage more cancer cells and less normal cells than NCTD. We conclude that both NOC15 and NC15 are safer anticancer drugs than NCTD in the treatment of Jurkat T cells relative to human normal lymphoblast. The SI can be further applied to the screening, developments, and applications of anticancer drugs in the future.

## 1 Introduction

The half maximal inhibitory concentration (IC_50_) is a measure of the effectiveness of a compound in inhibiting a specific biological or biochemical function by half. Similarly, the half maximal effective concentration (EC_50_) refers to the concentration of a compound which induces a half maximal effect after a specified exposure time. The 4-parameter logistic model (4PL) is often used to derive the IC_50_/EC_50_ from the sigmoid-shaped dose–response curve in the screening assays.^[1]^ The steepness of the linear portion of the curve is specified by the hillslope (HS) in the 4PL model.^[1]^ The IC_50_ and HS can be used to compare the therapeutic safety of anticancer drugs on cancer cells relative to normal cells.

Mylabris (*Mylabris phalerata* Pall.) is a species of blister beetle that has been used in traditional Chinese medicine in the treatment of hepatoma, breast cancer, colorectal cancer, and abdominal malignancy for more than 2000 years.^[2–5]^ One of the active compounds obtainable from Mylabris is cantharidin which has anticancer properties both in vitro and in vivo.^[6,7]^ Unfortunately, the clinical utility of cantharidin is restricted due to its nephrotoxicity and toxicity toward urinary system.^[8,9]^

A demethylated analog of cantharidin called norcantharidin (NCTD) is currently being used in China^[10]^ in the treatment of hepatoma,^[11]^ gallbladder carcinoma,^[12]^ leukemia,^[13]^ and colorectal carcinoma.^[14]^ Though NCTD has less nephrotoxicity^[5]^ and lower toxicity toward normal cells^[15,16]^ as compared to cantharidin, it is still not a satisfactory anticancer drug in terms of anticancer activity and toxicity. Thus, 2 analogs of NCTD were synthesized, namely, the *N*-farnesyloxy-norcantharimide (designated as NOC15) and *N*-farnesyl-norcantharimide (designated as NC15).^[17]^ Both NOC15 and NC15 have higher anticancer activities against hepatocellular carcinoma, bladder carcinoma, colorectal adenocarcinoma, and acute promyelocytic leukemia than NCTD,^[17]^ and can increase the survival days of mice, decrease the tumor weight, and retard the decrease in the weight of the spleen in a syngeneic mouse leukemia model.^[18]^

In our previous study, the anticancer activity ratio of drug X over drug Y toward cancer cells and the toxicity ratio of drug X over drug Y toward normal cells were defined as^[19]^ 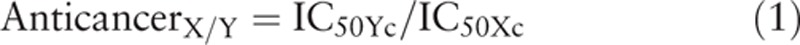 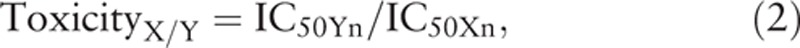


where the subscript “c” denotes cancer cells and the subscript “n” denotes normal cells, respectively. The “net effect” ratio can be employed to compare the therapeutic effects of 2 different anticancer drugs on cancer cells relative to their toxicity toward normal cells^[19]^ 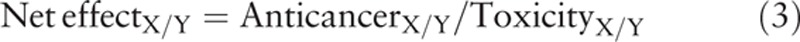


However, the relative safety of one anticancer drug against cancer cells relative to its toxicity toward normal cells was not given in the “net effect” ratio. Therefore, the aim of this study was to introduce a “safety index (SI)” to represent the therapeutic safety of one anticancer drug against cancer cells relative to its toxicity toward normal cells by using the 4PL model parameters.

## 2 Methods

### 2.1 Cells and cell culture

Both human normal lymphoblasts (HNL) and human leukemic Jurkat T cells (JKT) were purchased from the Bioresource Collection and Research Center (BCRC), Taiwan. The HNL and JKT cells were cultured in RPMI 1640 medium (GE Healthcare Life Sciences, Little Chalfont, UK) supplemented with 10% fetal bovine serum (FBS), 100 Unit/ml penicillin, and 100 μg/ml streptomycin at 37°C in a humidified 5% CO_2_ incubator.

Ethical approval of this study was waived because no human beings or animals were involved. Only cancer cells and normal cells were used in this study.

### 2.2 Cell viability assay

The cell viability assay of both HNL and JKT cells was performed in 96-well plates. A volume of 100 μl of cell suspension with 5×10^3^ cells/well in serum-free medium was inoculated in the wells and then preincubated in the incubator for 24 hours. Various concentrations of NCTD, NOC15, or NC15 were added to the wells. After 24 hours of incubation, the cell viability of HNL and JKT cells was assessed by using cell counting kit-8 (CCK-8, Sigma, St Louis, Missouri, USA). The colorimetric method was employed in the cell viability assay. The optical density of each well was measured at 450 nm using a spectrophotometer.

### 2.3 The 4PL model for cell viability curve

The IC_50_/EC_50_ of the drugs are often calculated using the non-linear regression analysis of the dose–response curve in the 4PL model^[20]^ 
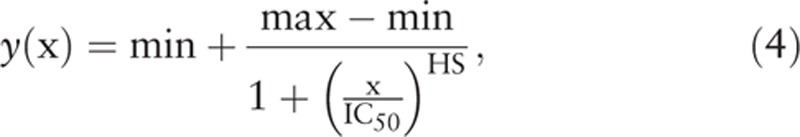


where y (x) is the cell viability as a function of drug concentration x, min is the lower asymptote of the dose–response curve or the lower plateau of y (x), max is the upper asymptote of the curve or the upper plateau of y (x). Let p be the percentage of inhibition in cell viability, and the corresponding inhibition concentration be denoted as IC_p_, we have then^[21]^ 
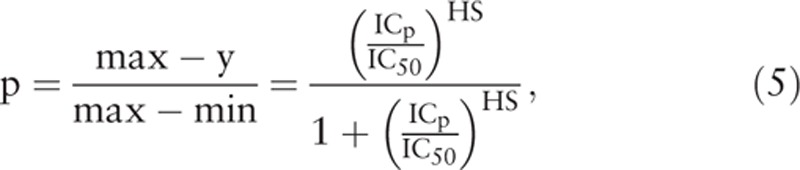
 
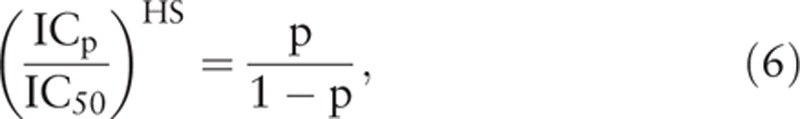
 
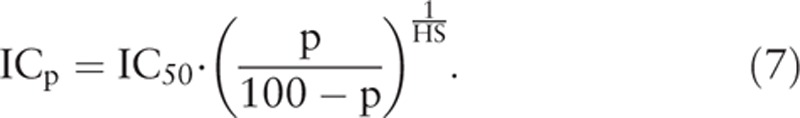


If we define the therapeutic range (TR) of a drug as the response interval between 10% and 90% inhibition, then the TR can be obtained from the above equations as follows 
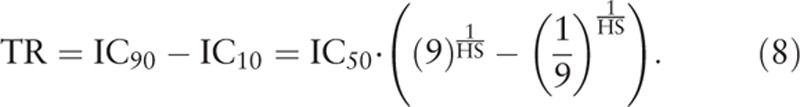


The change in cell viability due to the change in drug concentration is given by the first derivative of y (x) with respect to x 
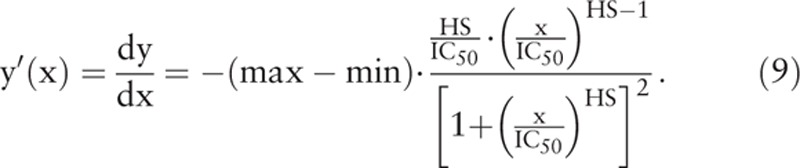


**Figure 1:**
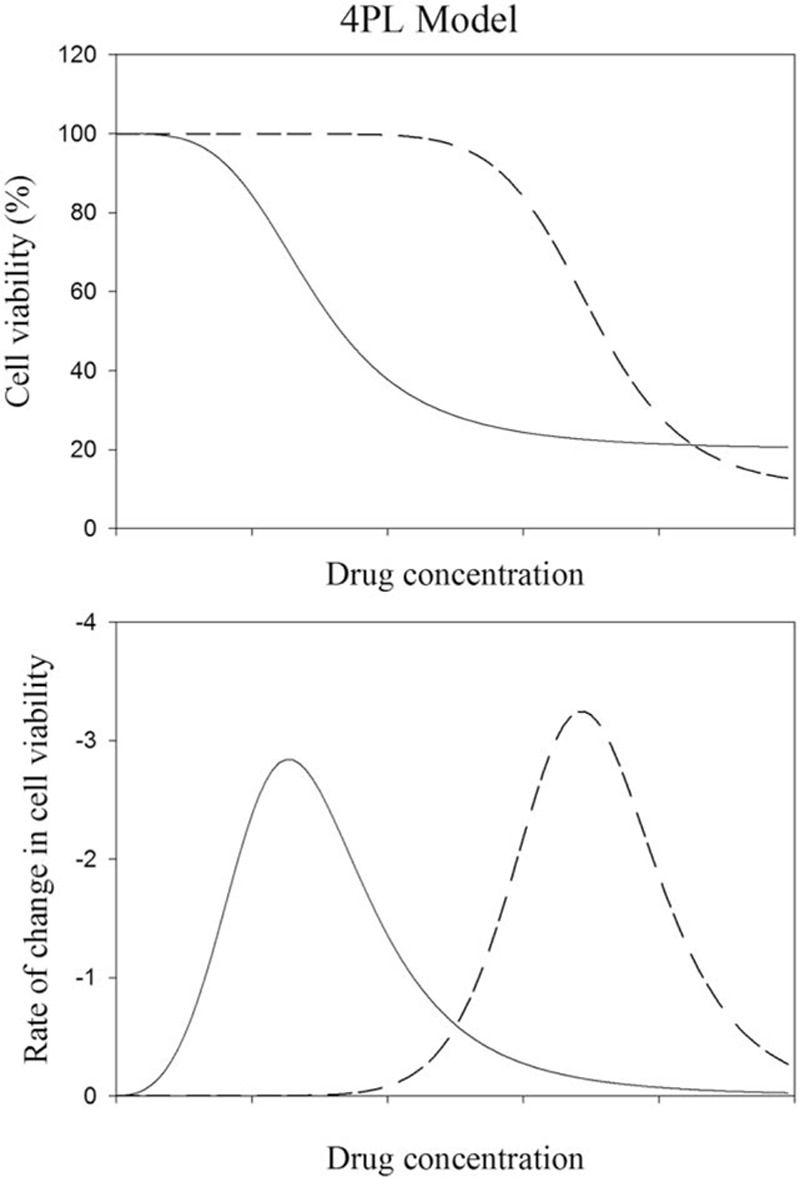
The curves of cell viability of cancer cells (solid line) and normal cells (dashed line) after the treatment with anticancer drug are shown in the upper panel. The corresponding curves for the rates of change in cell viability of cancer cells (solid line) and normal cells (dashed line) as the functions of drug concentration are shown in the lower panel. A wider separation between the cell viability curves of cancer cells (solid line) and normal cells (dashed line) after drug treatment means that the anticancer drug is safer because it can damage more cancer cells and less normal cells. The resemblance of the curves for the rates of change in cell viability as the functions of drug concentration in the lower panel to the elution peaks as the functions of elution time in HPLC suggests that the safety index (SI) can be defined similar to the resolution index (Rs) in HPLC to quantify the degree of separation between the curves of cell viability of cancer cells and normal cells.

Figure 1 shows 2 curves of cell viability of cancer cells (solid line) and normal cells (dashed line) after drug treatment, and 2 corresponding curves for the rates of change in cell viability of cancer cells (solid line) and normal cells (dashed line) as the functions of drug concentration. The curve for the rate of change in cell viability after drug treatment is skew-symmetric with peak value occurring at x_d_, which is the drug concentration where the rate of change in cell viability is the greatest. The x_d_ can be obtained by setting the second derivative of cell viability with respect to drug concentration equal to 0 
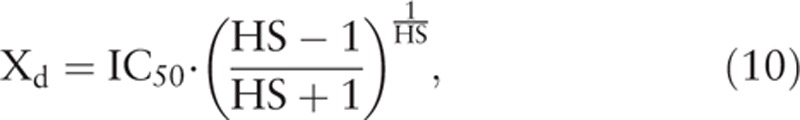


where the subscript “d” in x_d_ denotes “rapid decent.” The value of x_d_ is close to, but not the same as IC_50_. The skew-symmetric shape of y’(x) illustrates that the cell viability decreases gradually with drug concentration to the greatest extent at x_d_, and then returns gradually and asymptotically to zero. The maximum value or the peak height of y’(x) can be obtained by substituting x_d_ to the expression of y’(x) to give 
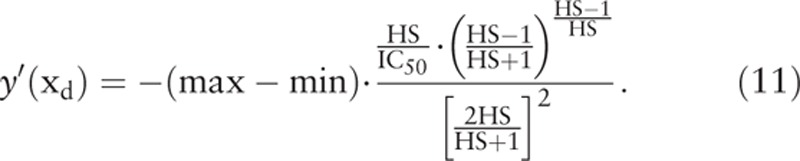


### 2.4 Safety index

In high performance liquid chromatography (HPLC), the resolution (Rs) is a measure of how well 2 chromatographic peaks are separated, or the ability of the column to resolve 2 analytes in 2 separate peaks. The standard 2-peak definition of resolution is^[22,23]^ 
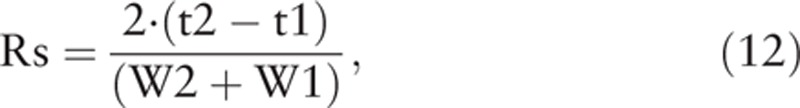


where t1 and W1 are the retention time and peak width of the first elution peak, and t2 and W2 are the retention time and peak width of the second elution peak, respectively. Since the skew-symmetric peak of y’(x) resembles the elution peak of HPLC, an index similar to the Rs in HPLC can be defined to quantify the separation of 2 skew-symmetric peaks of y’(x) in anticancer drug development (Fig. 1). The curves for the rates of change in cell viability as functions of drug concentration resemble the elution peaks as the functions of elution time in HPLC. Therefore, the SI in drug development can be devised similar to the Rs in HPLC to quantify the degree of separation between the curves of cell viability of different cells. In analogy to the Rs in HPLC, the SI is defined from IC_50_, HS, and TR as 
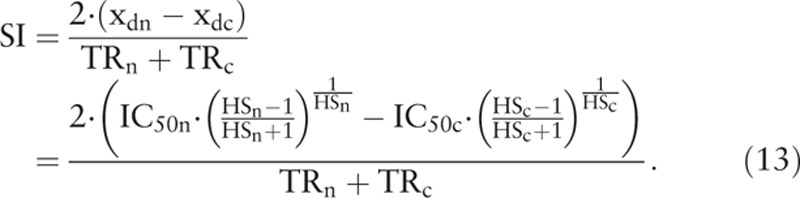


In the above definition, the x_d_ corresponds to the retention time t of the elution peak in HPLC, and the “TR” corresponds to the peak width of the elution peak in HPLC. Again, the subscript “n” denotes normal cells, and the subscript “c” denotes cancer cells, respectively. The right hand side of Eq. (13) is quite complex, and is hard to compute without the help of computer and suitable software. Since the difference between the x_d_ of 2 curves is what we need in the calculation of SI, rather than the absolute values of 2 x_d_, the (x_dn_−x_dc_) in Eq. (13) can be approximated by using the simpler (IC_50n_−IC_50c_) without much distortion. We have then the following simplified and practical expression for SI: 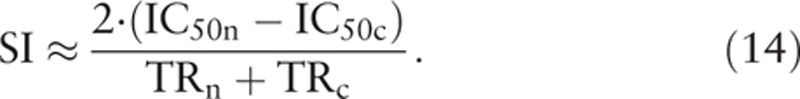


A larger SI means a greater separation between 2 cell viability curves. In the case of cancer cells versus normal cells, a larger SI means that the anticancer drug can damage more cancer cells and less normal cells. Thus, an anticancer drug with a larger SI should be a safer drug for anticancer therapy.

### 2.5 Anticancer ratio, the toxicity ratio, net effect ratio, and SI

To compare the anticancer ratio, the toxicity ratio, 4PL parameters, and SI of NCTD, NOC15, and NC15 on JKT and HNL cells, the JKT cells were treated with NCTD (0, 2.5, 5, 10, 20, 40, 80, and 160 μM), NOC15 (0, 0.25, 0.5, 1, 2, 4, 8, and 16 μM), and NC15 (0, 0.5, 1, 2, 4, 8, 16, and 32 μM) for 24 hours, respectively, while the HNL cells were treated with NCTD (0, 10, 20, 40, 80, 160, 320, and 640 μM), NOC15 (0, 2, 4, 8, 16, 32, 64, and 128 μM), and NC15 (0, 2, 4, 8, 16, 32, 64, and 128 μM) for 24 hours, respectively. The cell viability of the JKT and HNL cells was assessed by using the CCK-8 test. The IC_50_ and HS were calculated by using the 4PL regression model in the SigmaPlot 13 software package (SPSS Inc., Chicago, IL). The IC_10_, IC_90_, and TR were calculated using Eqs. (7) and (8), and the anticancer ratio, toxicity ratio, net effect ratio, and SI were calculated using Eqs. (1), (2), (3) and (14).

### 2.6 Statistical analysis

The experimental data were expressed as mean ± standard deviation (SD). Intragroup comparisons were performed by using repeated measures one-way ANOVA followed by Student–Newman–Keuls post hoc test. Between groups comparison was performed by using unpaired Student's *t*-test. A *P* < 0.05 was considered significantly different. All statistical analyses were performed by using SigmaPlot 13 software package.

## 3 Results

Table 1 shows that the anticancer ratio, toxicity ratio, and net effect ratio of NOC15 and NC15 over NCTD were all greater than 1, suggesting that the anticancer activity, toxicity, and net effect of NOC15 and NC15 were all greater than the corresponding values of NCTD. The net effect ratio of NOC15/NCTD and NC15/NCTD were 1.56 ± 0.52 and 1.35 ± 0.13, respectively. These data suggested that the net therapeutic effect of NOC15 and NC15 was about 1.56-fold and 1.35-fold that of NCTD, respectively.

**Table 1:**
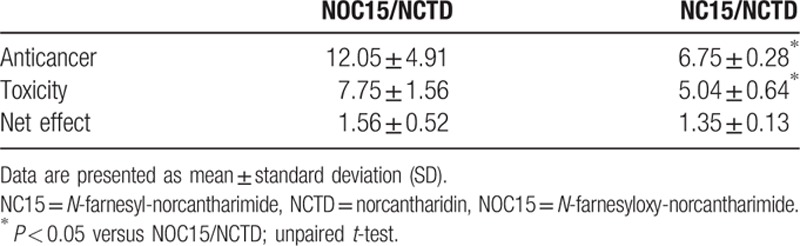
Comparisons of anticancer ratio, toxicity ratio, and net effect ratio between NOC15/NCTD and NC15/NCTD in JKT cells and HNL cells.

Table 1 also compares the anticancer ratio, toxicity ratio, and net effect ratio between NOC15/NCTD and NC15/NCTD. Both anticancer ratio and toxicity ratio of NOC15/NCTD were greater than those of NC15/NCTD, suggesting that the anticancer activity and toxicity of NOC15 relative to NCTD were greater than those of NC15 relative to NCTD. However, there were no significant differences in the net effect ratio between NOC15/NCTD and NC15/NCTD. This result suggested that the net therapeutic effect of NOC15 relative to NCTD was comparable to that of NC15.

Table 2 shows that the IC_50_ and IC_90_ of both NOC15 and NC15 were significantly smaller than the corresponding values of NCTD in both HNL cells and JKT cells. This result suggested that the anticancer activity of NOC15 and NC15 toward JKT cells and the toxicity of NOC15 and NC15 toward HNL cells were greater than those of NCTD. Moreover, the HS of NOC15 and NC15 was significantly greater than that of NCTD in HNL cells, and the HS of NC15 was significantly greater than that of NCTD in JKT cells, whereas the TR of NOC15 and NC15 was significantly smaller than that of NCTD, in both JKT and HNL cells. This result suggested that a subtle change in the concentration of NOC15 and NC15 would lead to a significant change in the anticancer activity of NOC15 and NC15 toward JKT cells and the toxicity toward HNL cells.

**Table 2:**
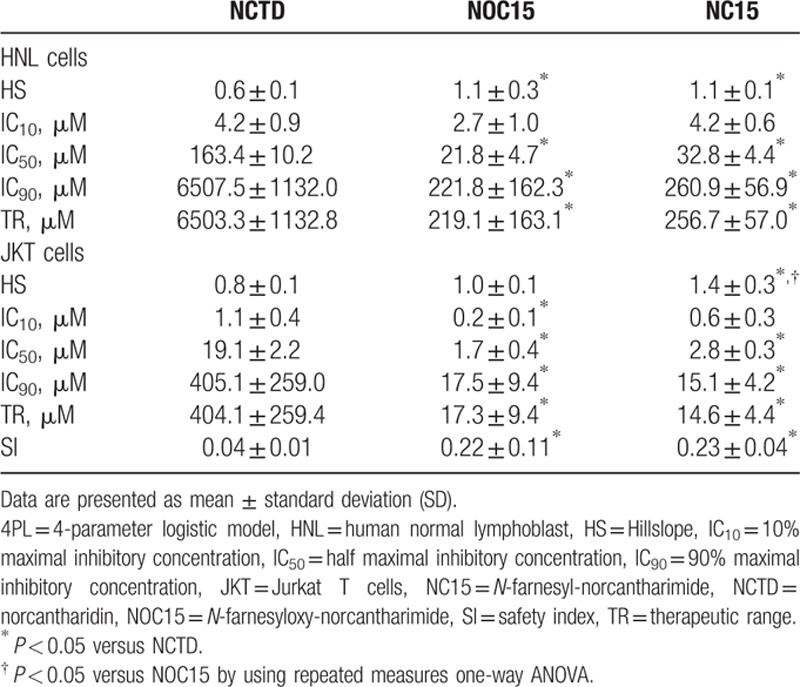
Comparisons of 4PL parameters and SI among NCTD, NOC15, and NC15 in JKT cells and HNL cells.

Table 2 also shows that the SI of NOC15 and NC15 was significantly greater than that of NCTD. This result indicated that NOC15 and NC15 might damage more cancer cells and less normal cells than NCTD. In other words, both NOC15 and NC15 might be safer than NCTD in the treatment of JKT cells, relative to HNL cells.

## 4 Discussion

The safety of an anticancer drug is one of the most important concerns of the physician in the treatment of cancer patients. To quantify the safety of an anticancer drug in the course of drug development, we devised the “safety index” in this study and compared the SI of 2 newly synthesized drugs, namely, the NOC15 and NC15, with the NCTD in the treatment of human leukemic JKT cells relative to HNL cells. We found that both NOC15 and NC15 had stronger anticancer activity toward JKT cells and higher toxicity toward HNL cells. However, both NOC15 and NC15 had larger SI than NCTD in the treatment of JKT cells relative to HNL cells. This means that the cell viability curves of JKT and HNL cells after treatment with NOC15 or NC15 were more separated than those after treatment with NCTD. A larger separation between the cell viability curve of cancer cells and that of normal cells after drug treatment indicates that the drug can damage more cancer cells and less normal cells, and is therefore a safer anticancer drug. Thus, both NOC15 and NC15 are better anticancer drugs against JKT cells than NCTD.

The IC_50_ and HS are usually used to represent the strength and sensitivity of the drug on the cells. The IC_50_ and HS of a drug on cancer cells can be compared with those on normal cells to assess the anticancer activity and toxicity of the drug. However, there are some situations where “more anticancer activity” implies “more toxicity.” Both NC15 and NOC15 fall into this situation. The safety of a drug in this situation is therefore difficult to evaluate by using IC_50_ and HS alone. To overcome this difficulty, we devised the SI in analogy to the Rs in HPLC to quantify the degree of safety of an anticancer drug. An anticancer drug with a larger SI relative to normal cells is therefore a safer drug in the treatment of cancer.

The “net effect” has been devised to compare the net therapeutic effects of 2 anticancer drugs^[19]^ so that the therapeutic effect of one anticancer drug relative to the other can be realized. Since the definition of “net effect” involves 2 drugs and 2 kinds of cell lines, it cannot be used to evaluate the therapeutic effect and toxicity of a single drug. To quantify the safety of one anticancer drug against cancer cells relative to its toxicity toward normal cells, other kind of index should be devised. Thus, we define the “safety index” in this study to quantify the safety of an anticancer drug against cancer cells relative to its toxicity toward normal cells. Both “net effect” and “safety index” can be used in the screening of anticancer drugs. However, different indices have different meanings and applications.

As shown in Table 2, the IC_90_ of NCTD on JKT cells (405.1 ± 259.0 μM) is much larger than the IC_10_ of NCTD on HNL cells (4.2 ± 0.9 μM). If we intend to kill 90% JKT cells by using 405.1 μM NCTD, then much more than 10% HNL cells will be damaged by NCTD. In contrast, the IC_90_ of NOC (17.5 ± 9.4 μM) and NC15 (15.1 ± 4.2 μM) on JKT cells are slightly greater than the IC_10_ of NOC15 (2.7 ± 1.0 μM) and NC15 (4.2 ± 0.6 μM) on HNL cells, respectively. If we intend to kill 90% JKT cells by using 17.5 μM NOC15 or 15.1 μM NC15, then slightly greater than 10% HNL cells will also be damaged by NOC15 and NC15. Thus, the comparison of the IC_90_ of an anticancer drug against cancer cells with the IC_10_ of the same anticancer drug against normal cells can give us important information about the safety of an anticancer drug in the treatment of cancer. This example illustrates the importance of the comparison between the IC_50_ and TR of an anticancer drug against cancer cells and the IC_50_ and TR of the same anticancer drug against normal cells at the same time. Based on this notion, the SI was constructed from the IC_50_ and TR of an anticancer drug against cancer cells and normal cells, and tested in the comparison of the safety among NCTD, NOC15, and NC15 in this study. Our result showed that the SI of either NOC15 or NC15 was greater than that of NCTD, suggesting that both NOC15 and NC15 are safer than NCTD in the treatment of JKT cells relative to HNL cells. Thus, the SI devised in this study is a concise index that can be used to quantify the degree of safety of an anticancer drug during the course of drug development.

Certain limitations of the present study need to be addressed. As this is a pilot study defining the SI and applying it to the comparison of therapeutic safety of 3 anticancer drugs on JKT cells relative to HNL cells, further studies concerning the comparisons of SI among more anticancer drugs against different kinds of cancer cells relative to different kinds of normal cells are needed so that the applicability and usefulness of SI can be realized. Second, the TR used in the calculation of SI was defined as the difference between IC_90_ and IC_10_ in this study; it can also be defined as the difference between IC_95_ and IC_5_, or else. What kind of definition is the best can be determined by more studies in the regard. Third, the SI defined in this study can be calculated in vitro using cancer cells and normal cells only. If the viability curves of cancer cells and normal cells in vivo can be obtained in the future, the in vivo SI can be defined accordingly. However, this task may not be easy to accomplish because the viability curves of cancer cells and normal cells in vivo are not easy to obtain due to the confounding effects of drug delivery, drug metabolism, blood supply, blood–brain barrier, variations in drug concentration at various tissues, pathophysiologic reactions of the host to the drug, and so forth.

In conclusion, based on the newly defined SI, both NOC15 and NC15 are safer anticancer drugs than NCTD in the treatment of JKT cells relative to HNL cells. The SI introduced in this study can be further applied to the screening, development, and applications of anticancer drugs in the future.

## References

[1] Richards FJ A flexible growth function for empirical use. *J Exp Botany* 1959; 10:290–300.

[2] Chen YN Chen JC Yin SC Effector mechanisms of norcantharidin-induced mitotic arrest and apoptosis in human hepatoma cells. *Int J Cancer* 2002; 100:158–165. 12115564 10.1002/ijc.10479

[3] Chen YJ Shieh CJ Tsai TH Inhibitory effect of norcantharidin, a derivative compound from blister beetles, on tumor invasion and metastasis in CT26 colorectal adenocarcinoma cells. *Anticancer Drugs* 2005; 16:293–299. 15711181 10.1097/00001813-200503000-00008

[4] Chen YJ Chang WM Liu YW A small-molecule metastasis inhibitor, norcantharidin, downregulates matrix metalloproteinase-9 expression by inhibiting Sp1 transcriptional activity in colorectal cancer cells. *Chem Biol Interact* 2009; 181:440–446. 19616522 10.1016/j.cbi.2009.07.004

[5] Wang GS Medical uses of mylabris in ancient China and recent studies. *J Ethnopharmacol* 1989; 26:147–162. 2689797 10.1016/0378-8741(89)90062-7

[6] Chen RT Hua Z Yang JL Studies on antitumor actions of cantharidin. *Chin Med J* 1980; 57:475–478. 6766849

[7] McCluskey A Ackland SP Bowyer MC Cantharidin analogues: synthesis and evaluation of growth inhibition in a panel of selected tumour cell lines. *Bioorg Chem* 2003; 31:68–79. 12697169 10.1016/s0045-2068(02)00524-2

[8] Tagwireyi D Ball DE Loga PJ Cantharidin poisoning due to “Blister beetle” ingestion. *Toxicon* 2000; 38:1865–1869. 10858524 10.1016/s0041-0101(00)00093-3

[9] Zhang L Sun X Zhang ZR An investigation on liver: targeting microemulsions of norcantharidin. *Drug Deliv* 2005; 12:289–295. 16188728 10.1080/10717540500176829

[10] Chen YJ Kuo CD Tsai YM Norcantharidin induces anoikis through Jun-N-terminal kinase activation in CT26 colorectal cancer cells. *Anticancer Drugs* 2008; 19:55–64. 18043130 10.1097/CAD.0b013e3282f18826

[11] Wu LT Chung JG Chen JC Effect of norcantharidin on N-acetyltransferase activity in HepG2 cells. *Am J Chin Med* 2001; 29:161–172. 11321474 10.1142/S0192415X01000186

[12] Fan YZ Fu JY Zhao ZM Effect of norcantharidin on proliferation and invasion of human gallbladder carcinoma GBC-SD cells. *World J Gastroenterol* 2005; 11:2431–2437. 15832413 10.3748/wjg.v11.i16.2431 PMC4305630

[13] Li JL Cai YC Liu XH Norcantharidin inhibits DNA replication and induces apoptosis with the cleavage of initiation protein Cdc6 in HL-60 cells. *Anti-Cancer Drugs* 2006; 17:307–314. 16520659 10.1097/00001813-200603000-00009

[14] Hill TA Stewart SG Sauer B Heterocyclic substituted cantharidin and norcantharidin analogues—synthesis, protein phosphatase (1 and 2A) inhibition, and anticancer activity. *Bioorg Med Chem Lett* 2007; 17:3392–3397. 17451951 10.1016/j.bmcl.2007.03.093

[15] Kok SH Hong CY Kuo MY Comparisons of norcantharidin cytotoxic effects on oral cancer cells and normal buccal keratinocytes. *Oral Oncol* 2003; 39:19–26. 12457717 10.1016/s1368-8375(01)00129-4

[16] Massicot F Dutertre-Catella H Pham-Huy C In vitro assessment of renal toxicity and inflammatory events of two protein phosphatase inhibitors cantharidin and nor-cantharidin. *Basic Clin Pharmacol Toxicol* 2005; 96:26–32. 15667592 10.1111/j.1742-7843.2005.pto960104.x

[17] Wu JY Kuo CD Chu CY Synthesis of novel lipophilic N-substituted norcantharimide derivatives and evaluation of their anticancer activities. *Molecules* 2014; 19:6911–6928. 24865603 10.3390/molecules19066911 PMC6271113

[18] Chang MC Tsai ET Wu JY N-farnesyloxy-norcantharimide and N-farnesyl-norcantharimide inhibit progression of leukemia and increase survival days in a syngeneic mouse leukemia model. *Anticancer Drugs* 2015; 26:508–517. 25588161 10.1097/CAD.0000000000000210

[19] Chang MC Wu JY Liao HF N-Farnesyloxy-norcantharimide inhibits progression of human leukemic Jurkat T cells through regulation of mitogen-activated protein kinase and interleukin-2 production. *Anticancer Drugs* 2015; 26:1034–1042. 26288134 10.1097/CAD.0000000000000284 PMC4588604

[20] Sebaugh JL Guidelines for accurate EC50/IC50 estimation. *Pharm Stat* 2011; 10:128–134. 22328315 10.1002/pst.426

[21] GraphPad Prism v4, GraphPad Software, San Diego, CA, USA.

[22] Asbury GR Hill HH Evaluation of ultrahigh resolution ion mobility spectrometry as an analytical separation device in chromatographic terms. *J Microcol Sep* 2000; 12:172–178.

[23] Kazakevichy Y Lobrutto R HPLC for Pharmaceutical Scientists. 2007; New Jersey: John Wiley & Sons, Inc, 32.

